# Entropy generation of MHD flow of sodium alginate (C_6_H_9_NAO_7_) fluid in thermal engineering

**DOI:** 10.1038/s41598-021-04655-0

**Published:** 2022-01-13

**Authors:** T. N. Abdelhameed

**Affiliations:** 1 Basic Engineering Sciences Department, College of Engineering, Majmaah University, Majmaah, 11952 Saudi Arabia; 2 Mathematics Department, Faculty of Science, Beni-Suef University, Beni-Suef, 62514 Egypt

**Keywords:** Engineering, Mathematics and computing, Physics

## Abstract

In this paper, impacts of magnetic field and porosity on the entropy generation of sodium-alginate (C_6_H_9_NaO_7_) fluid are studied. C_6_H_9_NaO_7_ is taken over a moving and heated vertical wall. Heat transfer is due to free convection. Initially, the problem is formulated in the form of PDEs along with physical conditions and then written in non-dimensional form. Problem is solved via Laplace transform and expression in analytical form is established for temperature and velocity field. The related relations for entropy generation and Bejan number and entropy generation are also examined. Nusselt number and Skin-friction are calculated and plotted in graphs. For numerical computations, a finite difference scheme is used using MATLAB software. The results in tables and graphs are discussed for embedded parameters. It is found that the magnetic field and porosity have strong influence on velocity, entropy generation and Bejan number. For greater Hartman number, entropy generation magnitude is greater compared to the Bejan number, conversely, this variation in Bejan number is more efficient. The porosity effect showed that if the medium is more porous, the entropy generation can decreases 50% when porosity increase from Ka = 1 to Ka = 2, however the Bejan number increases.

## Introduction

The study in this article covers many implementations in nanotechnology, electrical and biomedicine, biotechnology, drug distribution, chemotherapy, food manufacturing, and numerous industries. The result of second Law of thermodynamics is entropy generation^1^, which expresses that when the whole system is in the stable situation or under the activity of reversible reactions, at that point the measure of total entropy perseveres in reversible (reverse) processes, accordingly, the total entropy constantly upsurges. The phenomenon of heat transfer through a chemically reacting fluid, friction, and mixing a finite temperature are the factors of irreversibility in a system which is called as entropy generation (EG). Additionally, the (EG) two sections (a) thermal irreversibility and (b) losses due to frictional forces. The idea of (EG) was given by Clausius^2^. Bejan^3^, provided a detailed note on (EG) in fundamental convective heat transfer. After that Bejan^4^ investigated (EG) rate of heat transfer using investigation of second law of thermodynamics. Bejan^5^, examined heat transfer and thermal design using second-law analysis. Bejan^6^ utilized the strategy for thermodynamic streamlining of limited size systems and limited time processes to investigate the (EG) minimization. In another paper, Revellin et al.^7^ elucidated a number of applications of (EG) analysis in saturated two phase flow. Soomro et al.^8^ determined the solutions of (EG) where they inspected the influence of magneto-hydrodynamics (MHD) on carbon nanotubes water-based nanofluid. Irreversible phenomenon of the system is due to Ohmic dissipation flow of heat and friction factors are included in the (EG) rate. Salas et al.^9^ analyzed MHD (EG) role in the induction devices like pumping phenomenon and electric generator. Rashidi et al*.*^10^ discussed the analysis of (EG) in a study MHD flow through a rotating disk with porous medium using the second law of thermodynamics. They mentioned that MHD flow has numerous applications in MHD energy generators and is also used in the conversion mechanism for space vehicles. Akbar et al*.*^11^ analyzed (EG) for a CNT suspension nanoliquid in vertical channel with peristalsis and the (EG) number. Ishaq et al.^12^ examined the unsteady thin film flow over a stretching sheet along with (EG).In another paper, Ishaq et al.^13^, analyzed the (EG) on two-dimensional Eyring-Powell nanofluid flow over a stretching sheet with the impact of mass and heat transfer. Darbari et al*.*^14^, studied the (EG) in fluid flow between a bounded channel. Bhatti et al*.*^15^ calculated the results of (EG) for the MHD flow of Carreaunan fluid upon a stretching sheet. Abdollahzadeh et al.^16^ calculated (EG) in thermal radiative loading of structures with separate radiators.Some recent interesting studies related to (EG) and nanofluid can be found in^17–25^ and the references therein.

Mahian et al.^26^ examined (EG) for the flow of water based alumina nanoliquid. They considered the impact of duct friction, particles-size, and various thermophysical approaches. Mahian et al.^27^ focused on the (EG) of fluid flow through a channel bounded with two revolving cylinders. Selimefendigil et al.^28^ numerically studied the (EG) for the nanoliquid flow in a tricked trapezoidal cavity with the consideration of MHD. Qinget al.^29^ specifically focused on (EG) in MHD flow of Casson nanoliquid upon a porous surface. Mahian et al.^30^ reported a comprehensive overview on the (EG) in flow of nanoliquids. Ellahi et al.^31^ reported an interesting problem (EG) for Cu–H2O nanofluid with main focus on shape effects of nanosized particles. Sheikholeslami et al.^32^ used the Lattice Boltzmann simulation method for the heat transfer enhancement in nanofluid together with entropy generation. Saqib et al.^33^ examined an interesting problem of entropy generation where they considered a fractional model of nanofluid in different types of fractionalized nanofluids. They obtained exact solutions for the considered models. M. M. Rashidi et al.^34^ simulated energy change of a hybrid Al_2_O_3_-Cu-H_2_O inside a lid-driven heated square cavity, their results display an expansion of heat removal by means of nanoparticles. Khan et al.^35^ performed an exact analysis and studied the (EG) in MHD flow over a flat plate such that the plate has wall shear stress. Bhatti et al.^36^ studied (EG) on MHD Eyring–Powell nanoliquid over a porous stretching surface. Li et al.^37^ provided a suitable investigation on (EG) of nanofluid in a heat exchanger. Abdelhameed et al.^38–42^ obtained exact solutions for velocity and temperature by using the Laplace transform technique. Rashidi et al.^43^ investigate the effect of height and roughness geometry on the condensation proprieties in rough and smooth nanochannels, their results show that when the roughness height increases the flow is more affected by condensation.

To overcome this exertion, entropy generation plays a prominent role in dissecting such situations. Recent developments in modelling of entropy generation for dissipative cross material with a quartic auto catalyst were reported. Entropy generation in the MHD peristalsis nanofluid within a porous medium was studied. Many researches were devoted to examining entropy generation in different non-linear flow and heat transform problems^30–35^. The above literature shows that several problems on entropy generation are done. However, entropy generation for MHD C_6_H_9_NaO_7_ fluid over an accelerated vertical plate embedded in a porous medium is not yet done. Being motivated by the above-mentioned discussions, the present work focuses on the entropy generation of MHD non-Newtonian Casson nanofluids in a vertical plate with the presence magnetic fields will be tackled.

Therefore, this article aims to study this aspect of entropy generation. The problem is first modelled and then dimensionless analysis is used to get a transformed system and then to solve it using Laplace transform technique. Equations for Bejan number and entropy generation are first developed in then outcomes are calculated numerically using finite difference scheme and assessed.

## Problem descriptions

**Figure 1:**
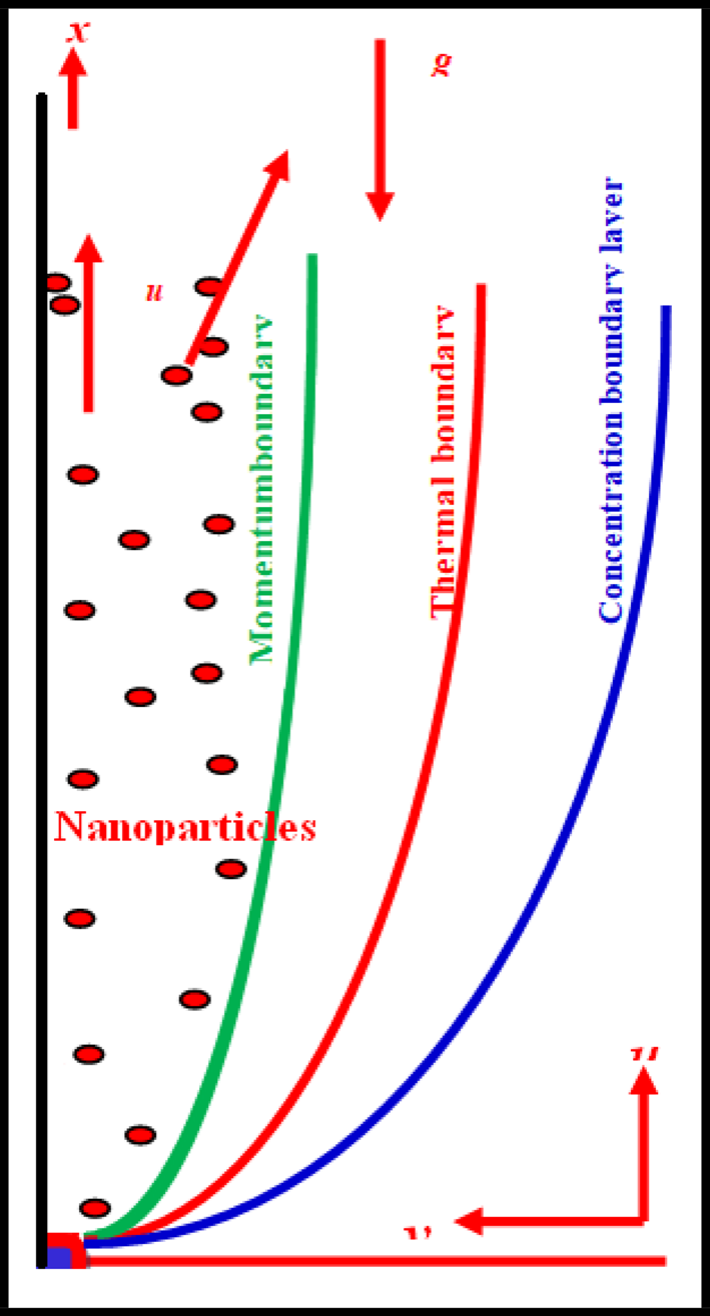
Physical description of the problem.

A Casson fluid with incompressible, unsteady mixed-convection flow next to an infinite vertical plate is considered. For $\tau \le 0$, the fluid and plate are considered to be stationary with surrounding temperature $\theta_{\infty }$. For $\tau = 0^{ + }$, the plate begins to slide with variable motion of $v\left( {0,\tau } \right) = A\tau$,in x-direction and the fluid at the plate raised to $\theta \left( {0,\tau } \right) = \theta_{w}$. The mixed convection occurs at this stage due to the change in temperature and velocity of the plate. The governing equations for the present flow regime are given by^17,37,44,45^. The physical model of the present article is shown in Fig. 1.

1 $$\rho \frac{\partial v(\eta ,\tau )}{{\partial \tau }} = \mu \left( {1 + \frac{1}{\beta }} \right)\frac{{\partial^{2} v(\eta ,\tau )}}{{\partial \eta^{2} }} + \rho g\beta_{\theta} (\theta (\eta ,\tau ) - \theta_{\infty } ) - \delta B_{0}^{2} v(\eta ,\tau ) - \frac{\mu \varphi }{{K_{p} }}v(\eta ,\tau )$$

2 $$\rho c_{p} \frac{\partial \theta (\eta ,\tau )}{{\partial \tau }} = k\frac{{\partial^{2} \theta (\eta ,\tau )}}{{\partial \eta^{2} }},$$

These are related to the subsequent physical initial and boundary conditions.

3 $$\left. {\begin{array}{*{20}l} {V\left( {\eta ,0} \right) = 0,\,} \hfill & {\theta \left( {\eta ,0} \right) = \theta_{\infty } } \hfill \\ {v\left( {0,\tau } \right) = A\tau ,} \hfill & {v\left( {\infty ,\tau } \right) = 0} \hfill \\ {\theta \left( {0,\tau } \right) = \theta_{w} ,\,} \hfill & {\theta \left( {\infty ,\tau } \right) = \theta_{\infty } } \hfill \\ \end{array} } \right\},$$

where v, θ, μ, ρ, g, $\beta_{\theta }$, k and $c_{p}$ are respectively the velocity, temperature, dynamic viscosity, density, gravitational acceleration, volumetric thermal expansion, thermal conductivity and heat capacitance of the fluid.

For non-dimensionalization, the dimensionless variables are as below:

$$v^{*} = \frac{v}{{\left( {vA} \right)^{\frac{1}{3}} }},\, \, \eta^{*} = \frac{{\eta A^{\frac{1}{3}} }}{{\nu^{\frac{2}{3}} }},\, \, \tau^{*} = \frac{{\tau A^{\frac{2}{3}} }}{{\nu^{\frac{1}{3}} }},\,\,\,\,\theta^{*} (\eta ,\tau ) = \frac{{\theta - \theta_{\infty } }}{{\theta_{w} - \theta_{\infty } }},$$

where $\nu$, fluid kinematic viscosity, $\theta$, fluid temperature and $\tau$ time.

- The fluid considered incompressible, the flow considered unsteady with mixed-convection as well with an infinite vertical plate.
- Close the plate the fluid is considered to be stationary with surrounding constant temperature.
- The plate begins to slide with variable motion in x-direction.
- The mixed convection occurs at this stage due to the change in temperature and velocity of the plate.

Table1 shows Sodium Alginate proprieties.

**Table 1:** Sodium alginate proprieties.

Physical properties	Sodium alginate
Cp (J/kg K)	4175
β (K^−1^)	0.001 10^–5^
σ (Ω^−1^ m^−1^)	5.01 10^–6^
K (W/mK)	0.6376
Pr	6
ρ (kg/m^3^)	989

This yields the following form.

4 $$\frac{\partial v(\eta ,\tau )}{{\partial \tau }} = \left( {1 + \frac{1}{\beta }} \right)\frac{{\partial^{2} v(\eta ,\tau )}}{{\partial \eta^{2} }} + Gr\theta (\eta ,\tau ) - H_{a} v(\eta ,\tau ) - \frac{1}{{K_{a} }}v(\eta ,\tau ),$$

5 $$\Pr \frac{\partial \theta }{{\partial \tau }} = \frac{{\partial^{2} \theta }}{{\partial \eta^{2} }},$$

6 $$\left. \begin{gathered} V\left( {\eta ,0} \right) = 0,\,\,\,\,\,\,\,v\left( {0,\tau } \right) = \tau ,\,\,\,\,\,\,v\left( {\infty ,\tau } \right) = 0\, \hfill \\ \theta \left( {0,\tau } \right) = 1,\,\,\,\,\,\,\theta \left( {\infty ,\tau } \right) = 0\,\,\,\,\,\theta \left( {\eta ,0} \right) = 0\,\, \hfill \\ \end{gathered} \right\},$$

where $$Gr = \frac{{g\beta_{\theta } \Delta \theta }}{A},{\text{ H}}_{{\text{a}}} = \frac{\delta }{\rho }B_{0}^{2} A^{{ - \frac{2}{3}}} \upsilon^{\frac{1}{3}} , \, \frac{{1}}{{{\text{K}}_{{\text{a}}} }} = \frac{\phi }{{K_{p} }}A^{{ - \frac{2}{3}}} \upsilon^{\frac{4}{3}} ,{\text{ Pr}} = \frac{{\mu {\text{c}}_{{\text{p}}} }}{{\text{K}}}$$

### Entropy generation

To decrease the energy loss and improve the heat transfer, the relation of entropy generation for Eqs. (4–6) is defined by^3–5,12,13^.

7 $$E_{gen} = \frac{K}{{\theta_{\infty }^{2} }}\left( {\frac{\partial \theta }{{\partial \eta }}} \right)^{2} + \frac{\mu }{{\theta_{\infty } }}\left( {1 + \frac{1}{\beta }} \right)\left( {\frac{\partial v}{{\partial \eta }}} \right)^{2} + \frac{{\delta \beta_{0}^{2} }}{{\theta_{\infty } }}v^{2} + \frac{\mu \phi }{{K_{p} \theta_{\infty } }}v^{2} .$$

Using the non-similarity variable, $\partial \theta /\partial \eta = \Delta \theta A^{\frac{1}{3}} \nu^{{ - \,\frac{2}{3}}} \partial \theta^{*} /\partial \eta^{*}$ and $\partial v/\partial y = A^{\frac{2}{3}} \nu^{{ - \,\frac{1}{3}}} \partial v^{*} /\partial \eta^{*}$ are introduced and by utilizing into Eq. (7), which yields

8 $$N_{s} = \frac{{E_{gen} }}{{E_{0} }} = \left[ {\left( {\frac{\partial \theta }{{\partial \eta }}} \right)^{2} + \frac{{B_{r} }}{\Omega }\left( {1 + \frac{1}{\beta }} \right)\left( {\frac{\partial v}{{\partial \eta }}} \right)^{2} + \frac{{B_{r} }}{\Omega }H_{a} v^{2} + \frac{{B_{r} }}{\Omega }\frac{1}{{K_{a} }}v^{2} } \right],$$

where

$$N_{s} = \frac{{E_{gen} \nu^{\frac{4}{3}} \theta^{2}_{\infty } }}{{kA^{2/3} (\Delta \theta )^{2} }},\,Br = \frac{{\mu A^{\frac{2}{3}} \nu^{\frac{2}{3}} }}{k\Delta \theta },\,\,\Omega = \frac{\Delta \theta }{{\theta_{\infty } }} = \frac{{\theta_{w} - \theta_{\infty } }}{{\theta_{\infty } }}.$$

### Bejan number

In the literature, for the first time, Bejan introduced different aspects to optimize the efficiency of thermal procedures. He introduced Bejan's number which is defined as the proportion between entropy production of heat transfer and entire entropy production. The Bejan number is given by

9 $$B_{e} = \frac{{\frac{K}{{\theta_{\infty }^{2} }}\left( {\frac{\partial \theta }{{\partial \eta }}} \right)^{2} }}{{\frac{K}{{\theta_{\infty }^{2} }}\left( {\frac{\partial \theta }{{\partial \eta }}} \right)^{2} + \frac{\mu }{{\theta_{\infty } }}\left( {1 + \frac{1}{\beta }} \right)\left( {\frac{\partial v}{{\partial \eta }}} \right)^{2} + \frac{{\delta \beta_{0}^{2} }}{{\theta_{\infty } }}v^{2} + \frac{\mu \varphi }{{K_{p} \theta_{\infty } }}v^{2} }}$$

and

10 $$B_{e} = \frac{{\left( {\frac{\partial \theta }{{\partial \eta }}} \right)^{2} }}{{\left( {\frac{\partial \theta }{{\partial \eta }}} \right)^{2} + \frac{{B_{r} }}{\Omega }\left( {1 + \frac{1}{\beta }} \right)\left( {\frac{\partial v}{{\partial \eta }}} \right)^{2} + \frac{{B_{r} }}{\Omega }H_{a} v^{2} + \frac{{B_{r} }}{\Omega }\frac{1}{{K_{a} }}v^{2} }}$$

## Exact solutions through Laplace transform scheme

In the previous works, solutions of different mixed convection problems are calculated numerically or analytically and exact or closed form solutions are very limited. The closed form solutions of the present problematic are carried out by applying the Laplace transform method. By applying the Laplace transform to Eqs. (4–6), we get

11 $$q\overline{v}(\eta ,q) = \left( {1 + \frac{1}{\beta }} \right)\frac{{\partial^{2} \overline{v}(\eta ,q)}}{{\partial \eta^{2} }} + Gr\overline{\theta }(\eta ,q) - H_{a} \overline{v}(\eta ,q) - \frac{1}{{K_{a} }}\overline{v}(\eta ,q)$$

12 $$\overline{v}\left( {0,q} \right) = \frac{1}{{q^{2} }},\,\,\,\,\,\,\overline{v}\left( {\infty ,q} \right) = 0$$

13 $$\Pr q\overline{\theta }\left( {\eta ,q} \right) = \frac{{\partial^{2} \overline{\theta }(\eta ,q)}}{{\partial \eta^{2} }}$$

14 $$\overline{\theta } \left( {0,q} \right) = \frac{1}{q},\,\,\,\,\,\,\overline{\theta } \left( {\infty ,q} \right) = 0$$

Using Eq. (14), the solutions of Eq. (13) become:

15 $$\overline{\theta }\left( {\eta ,q} \right) = \frac{{e^{{ - \eta \sqrt {\Pr q} }} }}{q}$$

By applying Laplace inverse, Eq. (15) takes the form:

16 $$\theta \left( {\eta ,\tau } \right) = erfc\left( {\frac{{\eta \sqrt {\Pr } }}{2\sqrt \tau }} \right)$$

In the same way, the solution of Eq. (11) becomes

17 $$\overline{v}(\eta ,q) = \frac{1}{{q^{{^{2} }} }}e^{{ - \eta \sqrt \upsilon \sqrt {q + M} }} - \frac{a}{{q(q + a_{0} )}}e^{{ - \eta \sqrt \upsilon \sqrt {q + M} }} + \frac{a}{{q(q + a_{0} )}}e^{{ - \eta \sqrt {\Pr } \sqrt q }}$$

where

$$a = \frac{\upsilon \, Gr}{{\upsilon - \Pr }}, \, a_{0} = \frac{M \, \upsilon }{{\upsilon - \Pr }},{\text{ M}} = {\text{H}}_{{\text{a}}} + \frac{1}{{K_{a} }}, \, \upsilon = {1} + \frac{{1}}{\beta }.$$

With the inverse Laplace transform,

18 $$v(\eta ,\tau ) = v_{1} (\eta ,\tau ) + v_{2} (\eta ,\tau ) + v_{3} (\eta ,\tau )$$

where

19 $$\begin{gathered} v_{1} (\eta ,\tau ) = \frac{1}{2}\left[ {\left( {\tau - \frac{\eta }{2}\sqrt {\frac{\upsilon }{M}} } \right)e^{{ - \eta \sqrt {\upsilon M} }} erfc\left( {\frac{\eta }{2}\sqrt {\frac{\upsilon }{\tau }} - \sqrt {M\tau } } \right) + \left( {\tau + \frac{\eta }{2}\sqrt {\frac{\upsilon }{M}} } \right)e^{{\eta \sqrt {\upsilon M} }} erfc\left( {\frac{\eta }{2}\sqrt {\frac{\upsilon }{\tau }} + \sqrt {M\tau } } \right)} \right] \hfill \\ v_{2} (\eta ,\tau ) = \frac{a}{{a_{0} }}\left[ \begin{gathered} \frac{{e^{{ - a_{0} \tau }} }}{2}\left( {e^{{ - \eta \sqrt {\upsilon \left( {M - a_{0} } \right)} }} erfc \, \left( {\frac{\eta }{2}\sqrt {\frac{\upsilon }{\tau }} - \sqrt {\left( {M - a_{0} } \right)\tau } } \right) + e^{{\eta \sqrt {\upsilon \left( {M - a_{0} } \right)} }} erfc \, \left( {\frac{\eta }{2}\sqrt {\frac{\upsilon }{\tau }} + \sqrt {\left( {M - a_{0} } \right)\tau } } \right)} \right) \hfill \\ - \frac{1}{2}\left( {e^{{ - \eta \sqrt {\upsilon M} }} erfc \, \left( {\frac{\eta }{2}\sqrt {\frac{\upsilon }{\tau }} - \sqrt {M\tau } } \right) + e^{{\eta \sqrt {\upsilon M} }} erfc \, \left( {\frac{\eta }{2}\sqrt {\frac{\upsilon }{\tau }} + \sqrt {M\tau } } \right)} \right) \hfill \\ \end{gathered} \right] \hfill \\ v_{3} (\eta ,\tau ) = \frac{a}{{a_{0} }}\left[ {erfc \, \left( {\frac{\eta }{2}\sqrt {\frac{\Pr }{\tau }} } \right) - \frac{{e^{{a_{0} \tau }} }}{2}\left( {e^{{ - \eta \sqrt { - a_{0} \Pr } }} erfc \, \left( {\frac{\eta }{2}\sqrt {\frac{\Pr }{\tau }} - \sqrt { - a_{0} \tau } } \right) + e^{{\eta \sqrt { - a_{0} \Pr } }} erfc \, \left( {\frac{\eta }{2}\sqrt {\frac{\Pr }{\tau }} + \sqrt { - a_{0} \tau } } \right)} \right)} \right] \hfill \\ \end{gathered}$$

### Skin friction

The skin friction for Casson fluid is determined by

20 $$c_{f} = \left( {1 + \frac{1}{\beta }} \right)\left. {\frac{\partial v(\eta ,\tau )}{{\partial \eta }}} \right|_{\eta = 0}$$

### Nusselt number

Similarly, the Nusselt number which is basically the heat transfer rate can be written as

21 $$Nu = \left. {\frac{\partial \theta (\eta ,\tau )}{{\partial \eta }}} \right|_{\eta = 0}$$

## Numerical solutions via finite difference scheme with results and discussion

In this paper, the analysis of entropy generation (EG) for the flow of Casson fluid was investigated. EG is very valuable in the applications related to heat transfer like analyzing heat exchangers. The exact solutions of the present problem are achieved using Laplace transform and numerically via finite difference scheme. The software used for the computation analysis is MATLAB. The purpose of this part is to show the influence of different parameters which affect the fluid motion. Furthermore, the current section highlights the velocity, temperature, EG, and Bejan number through graphical analysis. Furthermore, we have chosen sodium-alginate as base fluid which is in the class of non-Newtonian fluid like Casson fluid. In all the graphs the Prandtl number (Pr) is considered as 13.09. The value of Pr is fixed and it can be calculated from the expression $\Pr = \mu c_{p} /k$, by incorporating the values of $\mu = 0.002;\,\,\,k = 0.6376\,\,{\text{and}}\,\,c_{p} = 4175$ we get the value of $\Pr = 13.09$.

**Figure 2:**
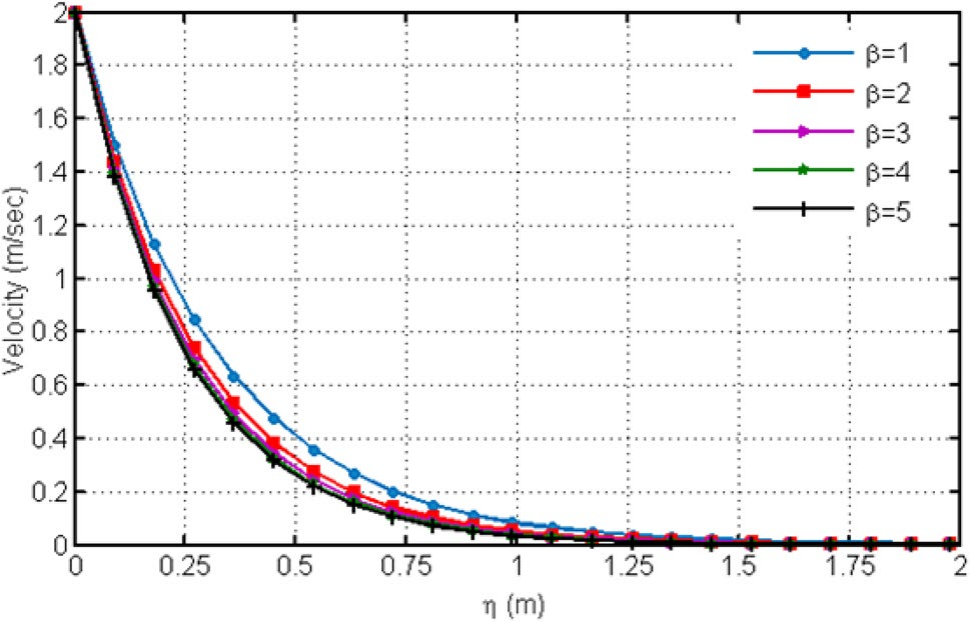
Variation in velocity profile for diverse values of C_6_H_9_NaO_7_ parameter $\beta$.

**Figure 3:**
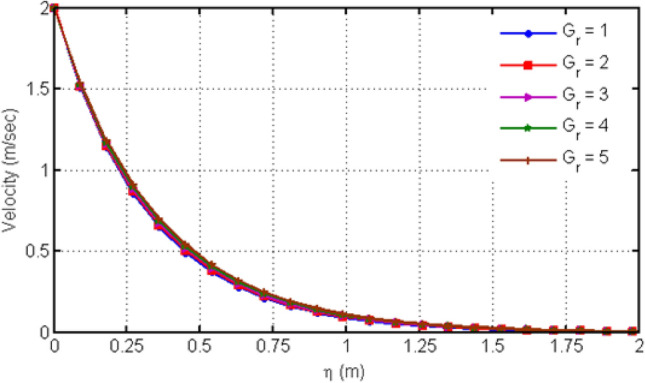
Variation in velocity profile for diverse values of $Gr$.

The C_6_H_9_NaO_7_ parameter $\beta$ (Casson-parameter) effect is shown in Fig. 2 which shows that the dual behavior is generated. The fluid velocity near to the plate increases while this behavior reverses away from the plate and shows decreasing effect for large values of β. The fact is that as increase in β reduces the boundary-layer thickness as a result velocity decreases. Figure 3 illustrates the variation of the velocity profile for different values of $Gr$.From the figure, it is clear that the velocity increases with the increase of the values of $Gr$. The increasing velocity with the increasing $Gr$ is obvious and it is due to the fact that $Gr$ enhances the buoyancy force, leading to the increase of fluid velocity. $Gr$ is the ratio of buoyancy with viscous force and expresses the thermal effect on the velocity of the fluids. Since we have considered laminar flow, where the range of $Gr$ is taken as $Gr < 10^{9}$ and for turbulent flow it can be taken as $Gr > 10^{9}$. In literature, many researchers have even taken $Gr = 0$ which reflects the absence of thermal effect, $Gr < 0$ which corresponds to the heating of the plate and cooling of the fluid and $Gr > 0$ which corresponds to cooling of the plate and heating of the fluid.

**Figure 4:**
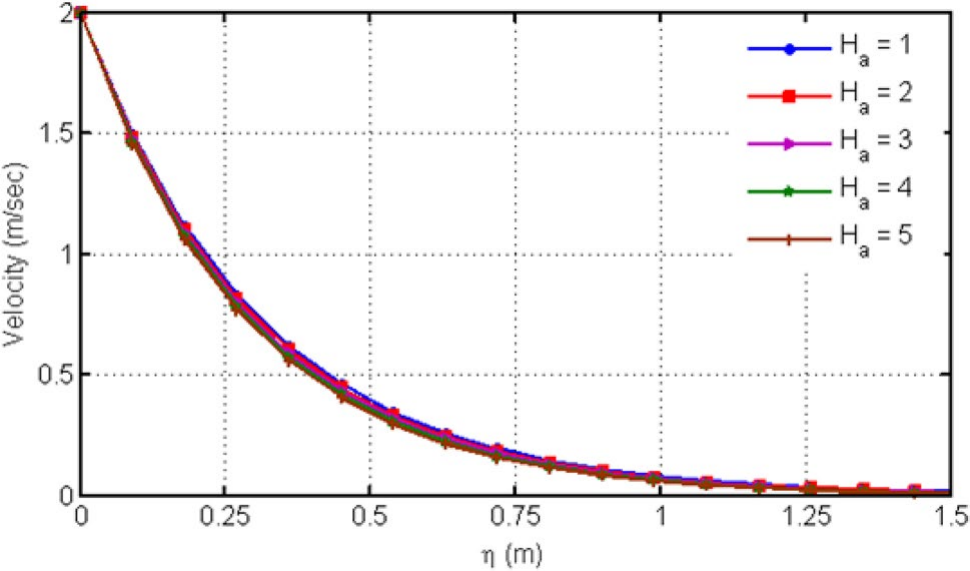
Variation in velocity profile for diverse values of Ha.

**Figure 5:**
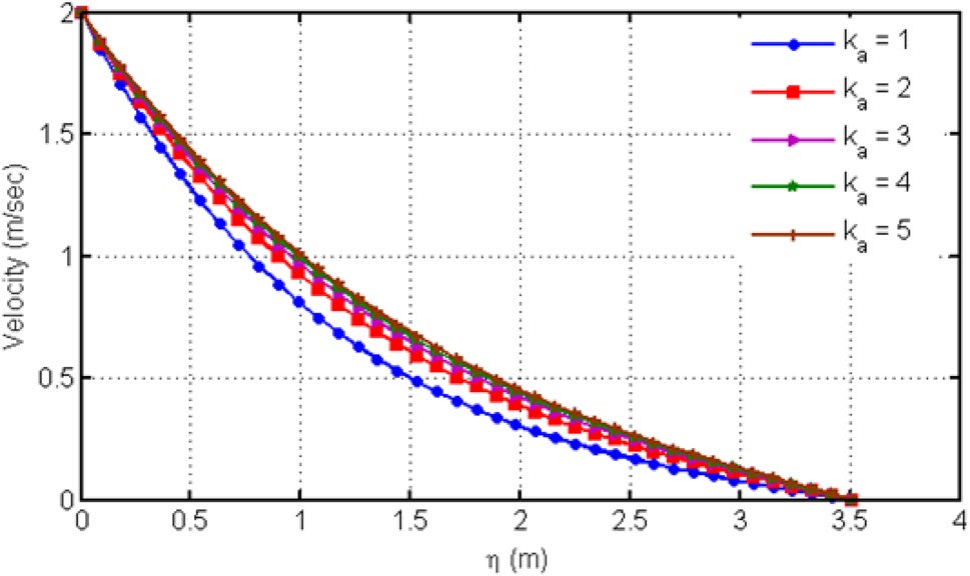
Variation in velocity profile for diverse values of porosity parameter Ka.

**Figure 6:**
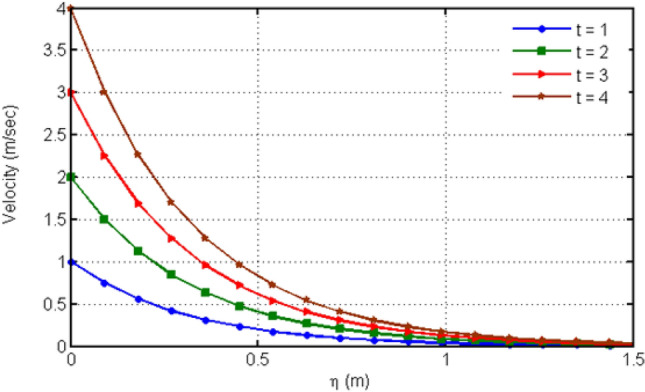
Variation in velocity profile for diverse values of time $t$.

**Figure 7:**
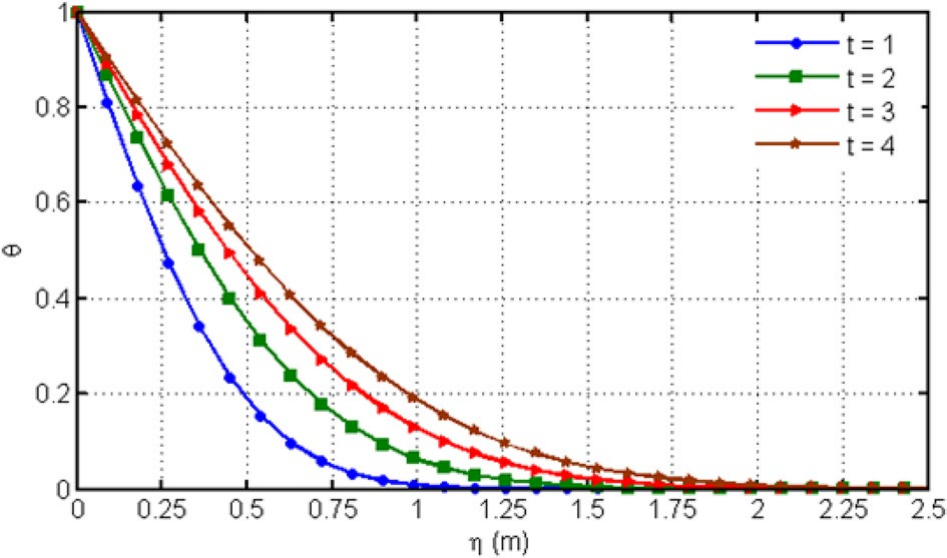
Variation in temperature profile for diverse values of time $t$.

Figure 4 displays the behavior of Hartman number on velocity profiles. From this figure, it can be detected that rising the values of Ha leads to a decrease in the velocity profile which is due to the fact that increasing Ha shows the Lorentz forces which are responsible of the fluid motion deceleration leading to the fact that velocity of the fluid is slow down. Figure 5 shows the impact of various values of porosity parameter Ka on velocity profile. Increasing Ka leads to the increase of the velocity profile. Figure 6 depicts the variation in velocity profile for different values of time $t$. As we have considered unsteady flow, this behavior can be seen from the graph and the fluid velocity increases when time increases. Actually, the fluid is assumed to be dependent on time, and consequently velocity rises with time. Figure 7 proves the influence of time on temperature profile. Augmenting time t leads to an increase in the temperature of the fluid. For large values of time, the temperature of the fluid becomes at its maximum.

**Figure 8:**
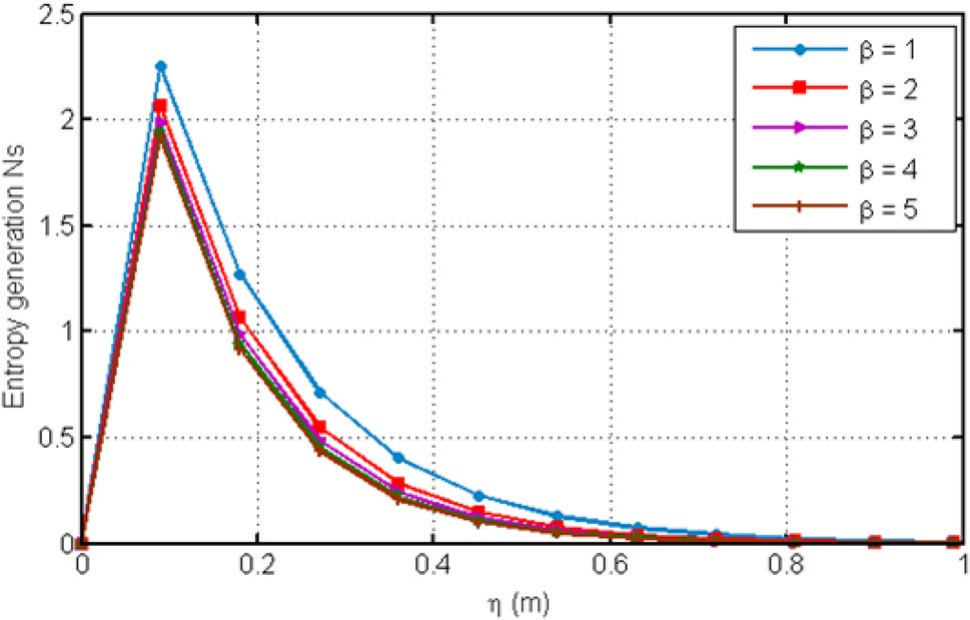
Variation in entropy generation for diverse values of C_6_H_9_NaO_7_ parameter $\beta$.

**Figure 9:**
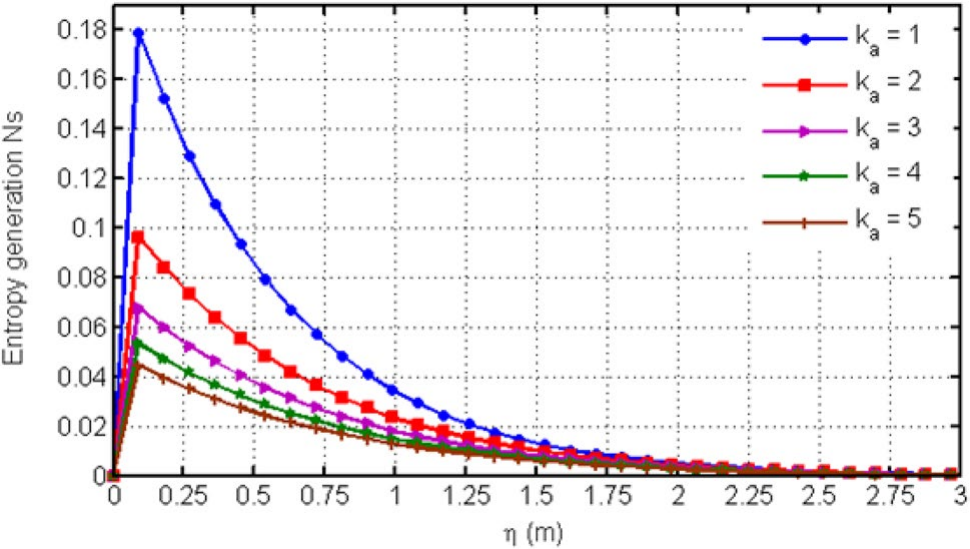
Variation in entropy generation for diverse values of porosity parameter Ka.

**Figure 10:**
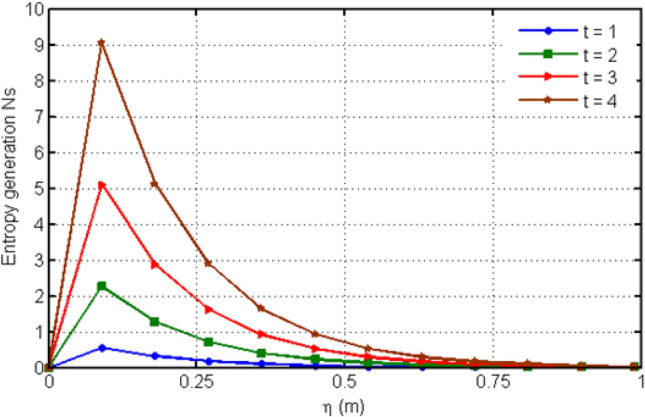
Variation in entropy generation for diverse values of time $t$.

**Figure 11:**
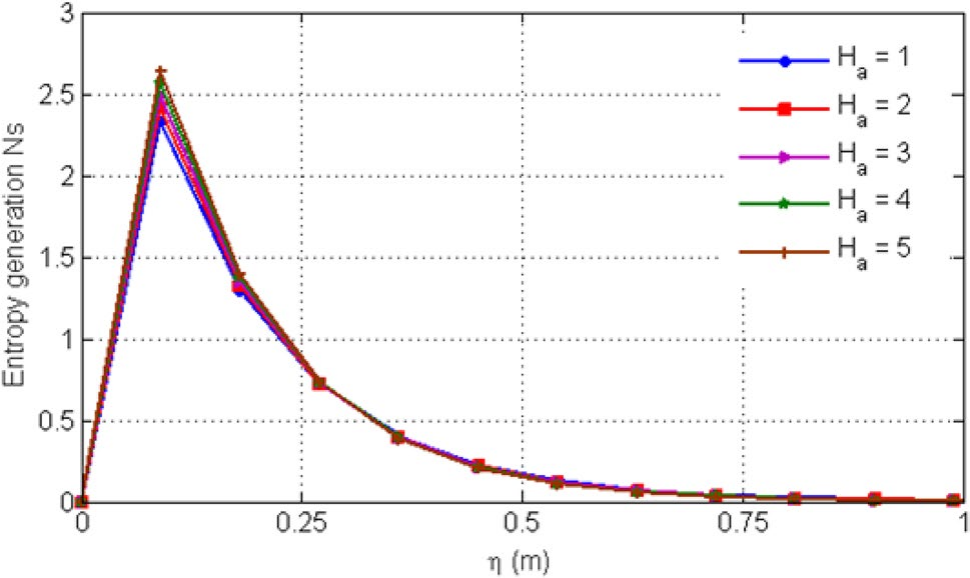
Variation in entropy generation for diverse values of Ha.

**Figure 12:**
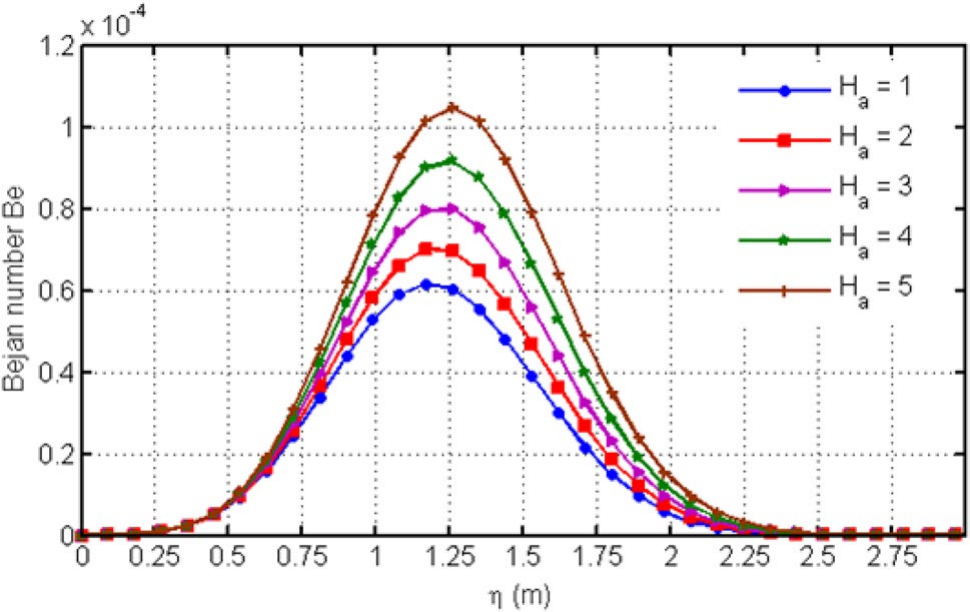
Variation in Bejan number for diverse values of Ha.

Figure 8 depicts variation in EG (Ns) for various values of Casson-parameter $\beta$. From the graph, it can be noticed that the EG decreases for larger values of $\beta$. Figure 9 illustrates the variation in EG for different values of porosity parameter Ka. For larger values of Ka, entropy generation is decreased. Figure 10 displays the variation in entropy generation for different values of time $t$. From the figure, we can see that increasing time increases the entropy generation. It means that entropy generation shows an increasing behavior which is due to the fact that we have considered an unsteady flow. Figure 11 depicts the variation in EG for different values of Ha. From the graph it can be seen that (EG) is directly proportional to Hartman number Ha. Increasing Ha results to an increase in the (EG). Figure 12 investigates the variation in Bejan number Be for different values of Ha. Increasing Ha leads to a rise in the Bejan number.

**Figure 13:**
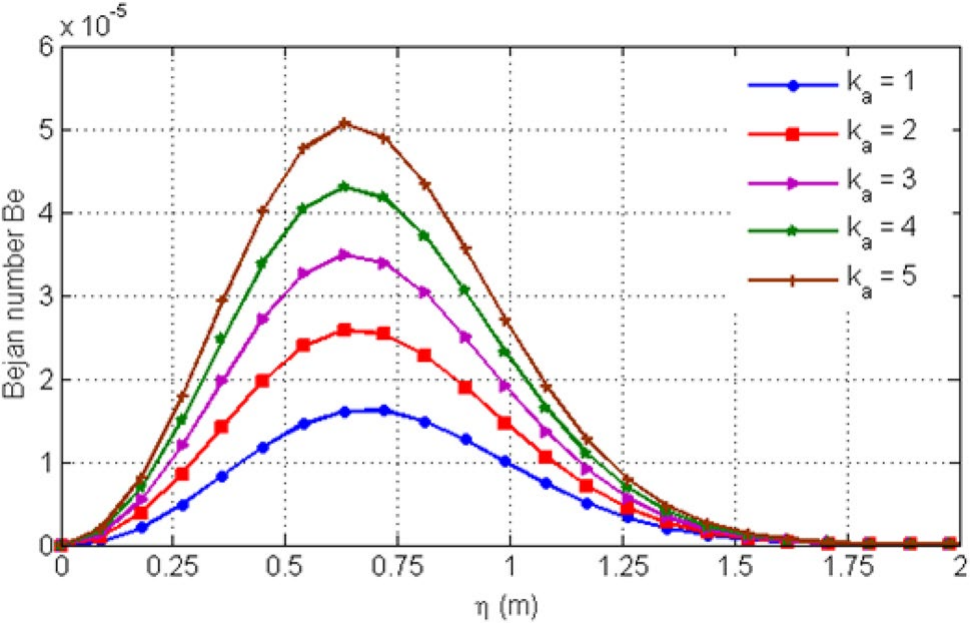
Variation in Bejan number for diverse values of porosity parameter Ka.

**Figure 14:**
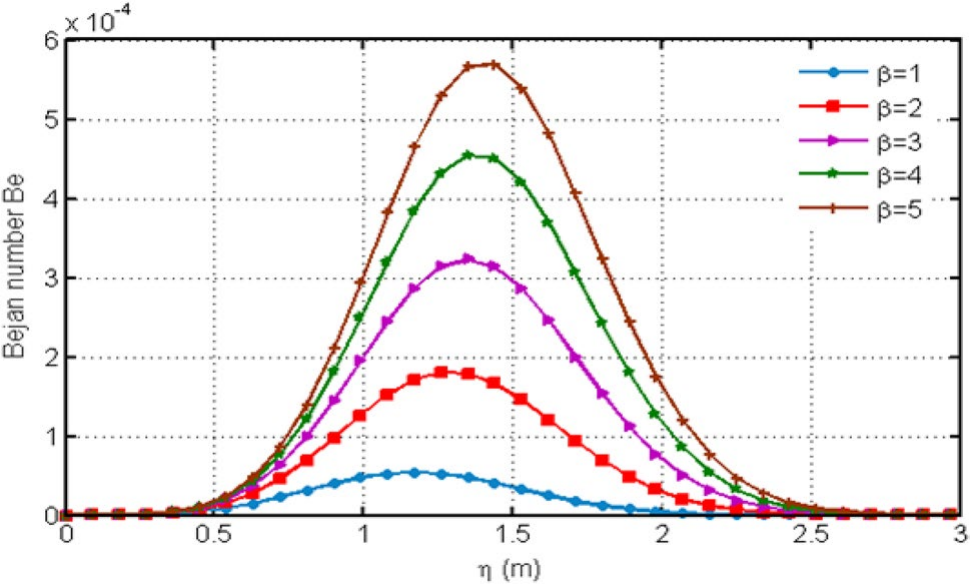
Variation in Bejan number for diverse values of C_6_H_9_NaO_7_ parameter $\beta$.

**Figure 15:**
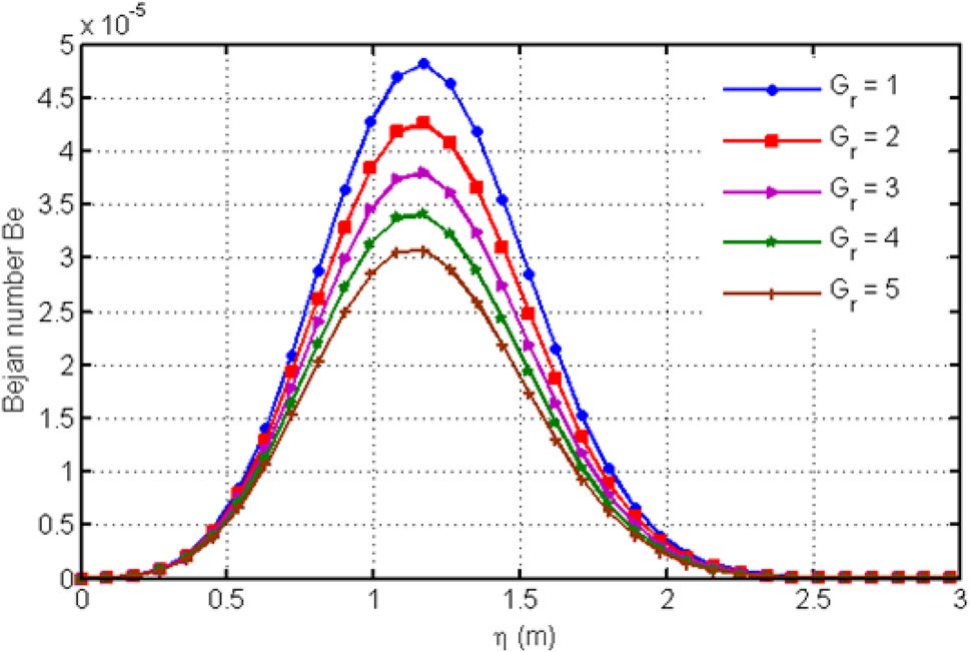
Variation in Bejan number for diverse values of $Gr$.

Figure 13 shows the variation in Bejan number for different values of porosity parameter Ka. Increasing Ka results to an increase in the Bejan number. The Casson-parameter $\beta$ variation is shown in Fig. 14 which illustrates that increasing the values of $\beta$ results to an increase in the Bejan number $Be$. This variation of Be is initially small but after some time Be shows greater variation for increasing values of $\beta$. Figure 15 highlights the influence of Bejan number Be for different values of $Gr$. From the graph, it is clear that increasing the values of $Gr$ leads to a decrease in Bejan number.

**Figure 16:**
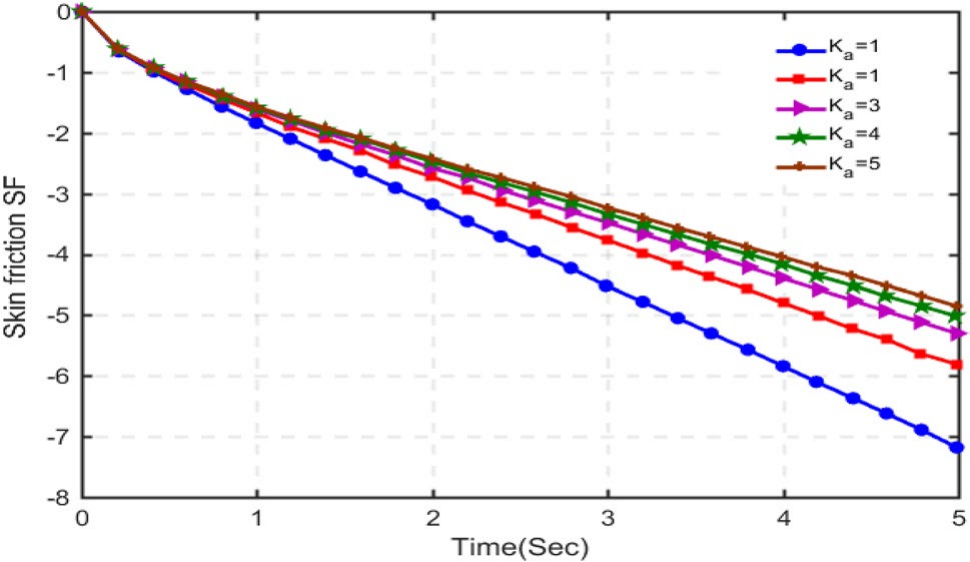
Variation in skin-friction for diverse values of Ka.

**Figure 17:**
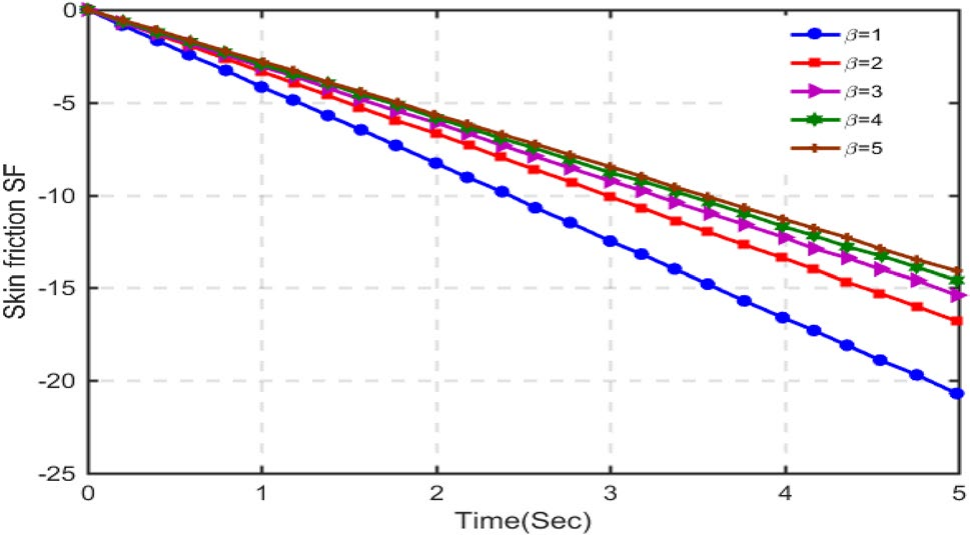
Variation in skin-friction for diverse values of C_6_H_9_NaO_7_ parameter $\beta$.

**Figure 18:**
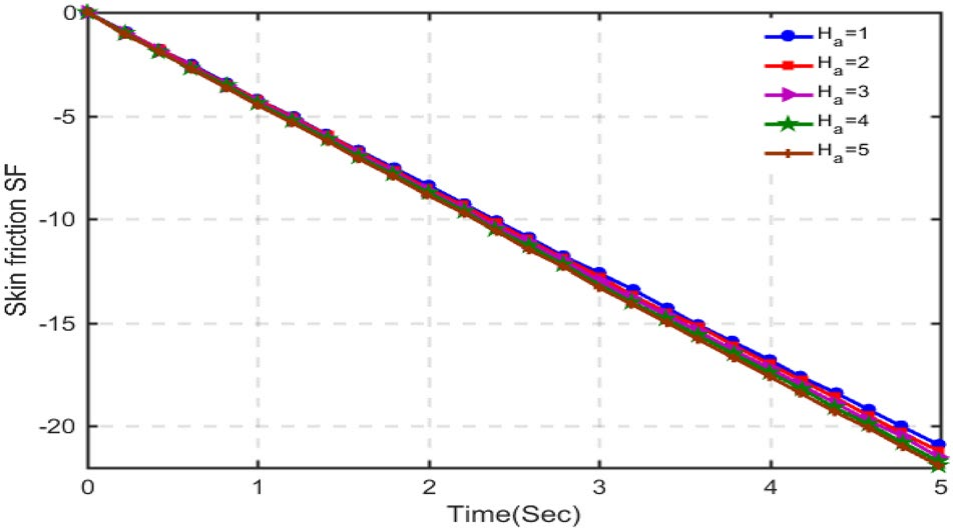
Skin-friction for different values of Ha.

**Figure 19:**
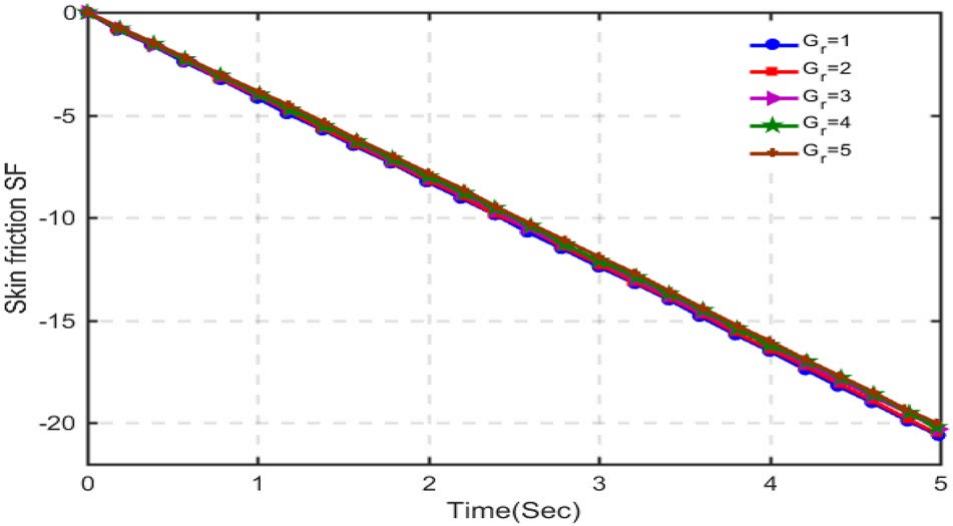
Skin-friction for different values of $Gr$.

The results for skin-friction are plotted in Figs. 16, 17, 18 and 19 for different values of porosity parameter ka, C_6_H_9_NaO_7_parameter $\beta$, Hartman number Ha and Grashof number $Gr$. Figure 16 shows that with increasing porosity parameter, skin-friction decreases as the values of skin-friction are in negative. This behavior is totally opposite to that of velocity. Increasing the porosity parameter, the pore size in the medium increases and hence the fluid can realize very little friction. Hence, the results are in accordance with the physical scenario. In Fig. 17, the results of skin-friction are plotted for different values of C_6_H_9_NaO_7_ parameter $\beta$. It is found that with increasing values of $\beta$, the skin friction decreases. This behavior is quite opposite to that velocity and is in accordance with physical situation. Figure 18 shows that the absolute value of skin-friction increases with increasing values of Hartmann number Ha. This is due to increasing Lorentz forces. Figure 19 indicates that with increasing Grashof number, the variation in skin-friction is not very visible, however, with a deep focus w can observe that the skin-friction increases with increasing Gr.

Table 2 shows our obtained solutions using finite difference scheme. The mathematical values assumed in table displays the velocity variation for the fluid entropy generation. These effects show that when growing values of $\beta$, velocity of EG decreases. Deviation in the velocity for diverse values of Ha are exposed in Table 3. we can observe that increasing Ha results to a reduction in the velocity magnitude. Table 4 shows variation in the velocity against ka. Increasing porosity results to an increase in the velocity. Physically, it is true since by increasing porosity, there will be an increase in the magnitude of velocity. Table 5 shows the variation of velocity for different values of Gr. Increasing Gr results to an increase in the magnitude of velocity.

**Table 2:** Finite difference scheme results of velocity for different values of $\beta$.

β	t	Ha	ka	Pr	Gr	η	v
1	2	0.05	0.05	14	0.05	0.5	0.1746
2	2	0.05	0.05	14	0.05	0.5	0.1337
3	2	0.05	0.05	14	0.05	0.5	0.1185
4	2	0.05	0.05	14	0.05	0.5	0.1106
5	2	0.05	0.05	14	0.05	0.5	0.1057

**Table 3:** Finite difference scheme results of velocity for different values of Ha.

Ha	t	β	Ka	Pr	Gr	η	v
1	2	1	0.05	14	0.05	0.5	0.1680
2	2	1	0.05	14	0.05	0.5	0.1615
3	2	1	0.05	14	0.05	0.5	0.1553
4	2	1	0.05	14	0.05	0.5	0.1495
5	2	1	0.05	14	0.05	0.5	0.1440

**Table 4:** Finite difference scheme results of velocity for different values of ka.

Ka	t	β	Ha	Pr	Gr	η	v
1	2	1	0.05	14	0.05	0.5	0.5607
2	2	1	0.05	14	0.05	0.5	0.5947
3	2	1	0.05	14	0.05	0.5	0.6070
4	2	1	0.05	14	0.05	0.5	0.6134
5	2	1	0.05	14	0.05	0.5	0.6173

**Table 5:** Finite difference scheme results for different values of Gr.

Gr	t	β	Ha	Pr	ka	η	v
1	2	1	0.05	14	0.05	0.5	0.1811
2	2	1	0.05	14	0.05	0.5	0.1880
3	2	1	0.05	14	0.05	0.5	0.1949
4	2	1	0.05	14	0.05	0.5	0.2017
5	2	1	0.05	14	0.05	0.5	0.2086

## Concluding findings

In this paper, the effects of MHD and porosity are studied on the entropy generation analysis of sodium-alginate (C6H9NaO7) fluid. The fluid motion is taken over a moving vertical plate which is heated from one side so that it causes heat transfer due to convection. The problem in terms of PDEs is formulated with some physical conditions. Exact analysis is then performed to obtain exact solutions using integrals transform method known as Laplace transform method. Outcomes for temperature, velocity, Nusselt number, Bejan number and entropy generation are summarized in tables and various schemes. The subsequent noteworthy remarks are deduced from this study.

- It is found that the magnetic field and porosity parameters have opposed impacts on velocity.
- Bejan number increases with increasing C_6_H_9_NaO_7_parameter, whereas entropy generation decreases.
- C_6_H_9_NaO_7_parameter has no effect on temperature and Nusselt number.
- For bigger values of Gr, the Effect on Bejan number is more evident when compared to entropy generation.
- For greater Hartman number, the entropy generation magnitude is greater compared to Bejan number, though, Bejan number variation is more efficient.
- Porosity effect showed that if the medium is more porous, the entropy generation decreases but Bejan number increases.
- The Nusselt number for small time is maximum near the plate as we move away from the plate this effect is reverses.

Our future work this investigation will be extended by utilizing variable thermal features, radiative influence and activation energy to enhancement heat and mass transfer mechanism for the same geometrical problem.

## Nomenclature

“v: Velocity of the fluid [ms^−1^]
$$\theta$$: Fluid temperature $$[{\text{K}}]$$
$$g$$: Gravitational acceleration $$[{\text{ms}}^{ - 2} ]$$
$c_{p}$: Specific heat at a constant pressure $[{\text{jkg}}^{ - 1} \,{\text{K}}^{ - 1} ]$
$Gr$: Thermal Grasshof number $( = \beta T_{w} )$
$k$: Fluid thermal conductivity $[{\text{Wm}}^{ - 2} \,\,{\text{K}}^{ - 1} ]$
$$Nu$$: Nusselt number $$[ - ]$$
$$\Pr$$: Prandtle number $$( = \mu c_{p} /k)$$
$\theta_{\infty }$: Fluid temperature distant to the plate $[{\text{K}}]$
$q$: Laplace transforms parameter
*A*: Random constant $$[{\text{ms}}^{ - 2} ]$$
*Ha*: Hartmann number
*K*_*a*_: Porosity parameter

## Greek symbols

$\nu$: Fluid kinematic viscosity $[{\text{m}}^{2} \,{\text{s}}^{ - 1} ]$
$$\rho$$: Fluid density $$[{\text{kg}}\,{\text{ms}}^{ - 3} ]$$
$$\mu$$: Dynamic viscosity $$[{\text{kgm}}^{ - 1} \,{\text{s}}^{ - 1} ]$$
$\beta_{\theta }$: The volumetric coefficient of thermal expansion $[{\text{K}}^{ - 1} ]$
$\beta$: The Casson fluid parameter
$B_{\gamma }$: The Brinkman number
$\Omega$: The dimensionless temperature function
